# Supplementary material for: A systematic review and meta-analysis on the rate of human schistosomiasis reinfection
Source: PLoS One. 2020 Dec 3;15(12):e0243224. doi: 10.1371/journal.pone.0243224 (PMC7714137; doi:10.1371/journal.pone.0243224)
Supplement: S1 Table — (DOCX) [file pone.0243224.s001.docx]

S1 Table. Electronic search strategy

| **No** | **Database** | **Date and time accessed** | **Search query** | **No of hits** | **Title and abstract accessed** |
| --- | --- | --- | --- | --- | --- |
|  | PUBMED | 31/08/2019, 08:49 am | (("allintitle"[All Fields] AND ("schistosomiasis"[MeSH Terms] OR "schistosomiasis"[All Fields] OR "schistosomiases"[All Fields])) OR ("schistosoma mansoni"[MeSH Terms] OR ("schistosoma"[All Fields] AND "mansoni"[All Fields]) OR "schistosoma mansoni"[All Fields]) OR ("schistosoma haematobium"[MeSH Terms] OR ("schistosoma"[All Fields] AND "haematobium"[All Fields]) OR "schistosoma haematobium"[All Fields])) AND ("reinfect"[All Fields] OR "reinfected"[All Fields] OR "reinfecting"[All Fields] OR "reinfection"[All Fields] OR "reinfections"[All Fields] OR "reinfects"[All Fields]) | 436 | 436 |
|  | HINARI  -Scholarly peer reviewed | 30/08/2019, 04:51 pm | schistosomiasis OR schistosoma mansoni OR schistosoma haematobium OR AND reinfection | 1371 | 500 |
|  | Google Scholar | 30/08/2019, 07:15 pm | "schistosomiasis OR schistosoma mansoni OR schistosoma haematobium" AND "reinfection" | 109 | 109 |
