# Supplementary material for: A systematic review and meta-analysis on the rate of human schistosomiasis reinfection
Source: PLoS One. 2020 Dec 3;15(12):e0243224. doi: 10.1371/journal.pone.0243224 (PMC7714137; doi:10.1371/journal.pone.0243224)
Supplement: S2 Table — (DOCX) [file pone.0243224.s002.docx]

S2 Table. Quality assessment results

| **SN** | **Author year** | **Quality criteria score** | | | | | | | | | |
| --- | --- | --- | --- | --- | --- | --- | --- | --- | --- | --- | --- |
|  |  | **A** | **B** | **C** | **D** | **E** | **F** | **G** | **H** | **I** | **Total** |
| 1 | Belizario 2008 | 1 | 1 | 1 | 1 | 1 | 1 | 1 | 1 | 1 | 9 |
| 2 | Chandiwana 1991 | 1 | 0 | 1 | 1 | 1 | 1 | 1 | 0 | 1 | 7 |
| 3 | de Lima 1993 | 1 | 1 | 1 | 1 | 1 | 1 | 1 | 1 | 1 | 9 |
| 4 | de Moira 2010 | 1 | 1 | 0 | 1 | 1 | 1 | 1 | 1 | 0 | 7 |
| 5 | Egesa 2018 | 1 | 1 | 0 | 1 | 1 | 1 | 1 | 0 | 1 | 7 |
| 6 | Favre 2015 | 1 | 1 | 1 | 1 | 1 | 1 | 1 | 1 | 1 | 9 |
| 7 | Friis 1997 | 1 | 1 | 1 | 1 | 1 | 1 | 1 | 1 | 1 | 9 |
| 8 | Garba 2013 | 1 | 0 | 1 | 1 | 1 | 1 | 1 | 1 | 1 | 8 |
| 9 | Gazzinelli 2017 | 1 | 0 | 1 | 1 | 1 | 1 | 1 | 0 | 1 | 7 |
| 10 | Gyoten 1992 | 0 | 0 | 1 | 1 | 1 | 1 | 1 | 0 | 0 | 5 |
| 11 | Haumson 2018 | 1 | 0 | 1 | 0 | 1 | 1 | 1 | 1 | 1 | 7 |
| 12 | Jiz 2009 | 1 | 1 | 1 | 1 | 1 | 1 | 1 | 1 | 1 | 9 |
| 13 | Kabuyaya 2017 | 1 | 1 | 0 | 1 | 1 | 1 | 1 | 1 | 1 | 8 |
| 14 | Lemos 2019 | 1 | 1 | 0 | 1 | 1 | 1 | 0 | 1 | 1 | 7 |
| 15 | Li 2000 | 1 | 1 | 0 | 1 | 1 | 1 | 1 | 1 | 1 | 8 |
| 16 | Mduluza 2001 | 1 | 1 | 1 | 1 | 1 | 1 | 1 | 0 | 1 | 8 |
| 17 | Makuvaza 2018 | 1 | 1 | 1 | 1 | 1 | 1 | 1 | 0 | 1 | 8 |
| 18 | Munisi 2017 | 1 | 1 | 1 | 1 | 1 | 1 | 1 | 1 | 1 | 9 |
| 19 | Mutapi 1999 | 1 | 1 | 1 | 1 | 1 | 1 | 1 | 1 | 1 | 9 |
| 20 | Nalugwa 2015 | 1 | 1 | 1 | 1 | 1 | 1 | 1 | 1 | 0 | 8 |
| 21 | Ofoezie 2000 | 0 | 1 | 0 | 0 | 1 | 1 | 1 | 1 | 1 | 6 |
| 22 | Olliaro 2011 | 1 | 1 | 1 | 0 | 1 | 1 | 1 | 1 | 1 | 8 |
| 23 | Reis 2006 | 1 | 1 | 1 | 1 | 1 | 1 | 1 | 1 | 1 | 9 |
| 24 | Satti 1996 | 1 | 1 | 0 | 1 | 1 | 1 | 1 | 1 | 1 | 8 |
| 25 | Senghor 2015 | 1 | 1 | 1 | 1 | 1 | 1 | 1 | 1 | 1 | 9 |
| 26 | Senghor 2016 | 1 | 1 | 1 | 1 | 1 | 1 | 1 | 1 | 1 | 9 |
| 27 | Webster 2012 | 1 | 1 | 1 | 1 | 1 | 1 | 1 | 0 | 1 | 8 |
| 28 | Woldegerima 2019 | 1 | 1 | 0 | 1 | 1 | 1 | 1 | 1 | 1 | 8 |
| 29 | Zhaosong 1997 | 1 | 1 | 1 | 0 | 1 | 1 | 1 | 0 | 1 | 7 |
| **Average** | | | | | | | | | | | 7.9 |

Note: A - appropriateness of sample frame to address the target population, B – appropriateness of the way used to sample study participants, C – adequateness of sample size, D – description of study subjects and settings, E – data analysis coverage in the identified sample, F – validity of method used to identify schistosomiasis reinfection rate, G – reliability of method used to measure schistosomiasis reinfection rate for all participants, H – appropriateness of statistical tests used in data analysis and I – adequateness of response rate
