# Supplementary material for: A systematic review and meta-analysis on the rate of human schistosomiasis reinfection
Source: PLoS One. 2020 Dec 3;15(12):e0243224. doi: 10.1371/journal.pone.0243224 (PMC7714137; doi:10.1371/journal.pone.0243224)
Supplement: S1 File — (DOC) [file pone.0243224.s004.doc]

**Review Protocol**

**Review title:** A systematic review and meta-analysis of the rate of schistosomiasis reinfection

1. **Background**
   1. Important characteristics

Human schistosomiasis infection occurs in water through skin penetration by infective larvae (cercariae) of the five species of Schistosoma (*Schistosoma haematobium, Schistosoma mekongi, Schistosoma intercalatum, Schistosoma mansoni* or *Schistosoma japonicum*) (1). Schistosomiasis is prevalent in tropical and subtropical areas, especially in poor communities without access to safe drinking water and adequate sanitation. It is estimated that at least 90% of those requiring treatment for schistosomiasis live in Africa. The disease affects people of all sex and age groups, but it is common in school children. Diagnosis is based on the identification of eggs in urine for urogenital schistosomiasis or stool for intestinal schistosomiasis*.* Point of care antigenic diagnostic kits also is available. Intestinal schistosomiasis can result in abdominal pain, diarrhea, blood in stool and in advanced cases enlargement of liver and spleen. Urinary schistosomiasis is characterised by haematuria, fibrosis of the bladder and kidney damage. Urinary schistosomiasis may also result to vaginal bleeding, pain during sexual intercourse and vulva nodules in women; it can induce pathology of seminal vesicles, prostate and infertility in men (2,3). The economic and health effects of schistosomiasis are considerable and the disease disables more than it kills. In children, schistosomiasis can cause anaemia, stunting and a reduced ability to learn. Chronic schistosomiasis may affect people’s ability to work and in some cases can result in death. The number of deaths due to schistosomiasis is difficult to estimate because of hidden pathologies such as liver and kidney failure, bladder cancer and ectopic pregnancies due to female genital schistosomiasis (2,4).

The interventions such as preventive chemotherapy (praziquantel), snail control, health education and improvement of water supply and sanitation facilities have been going on in order to prevent and control schistosomiasis (5,6). Large scale use of preventive chemotherapy has been very useful in the reduction of morbidity and mortality due to schistosomiasis. However, schistosomiasis transmission is not yet interrupted by the current control measures (6,7). It has also been observed that treatment does not avert subsequent infection; thus, if contact with infested water is continued, re-infection can take place relatively quickly (8). Several factors have been associated with the rapid schistosomiasis reinfection rate such as age, gender, sex, pre-treatment intensity, level of immunity and water contact behaviour attributed to economic, domestic and recreational activities (9–11). However the effects of these factors were shown to vary depending on the type of *Schistosoma* species and its geographical distribution

- 1. Relevance

Schistosomiasis remains a major global health problem. The morbidity and mortality due to schistosomiasis have decreased considerably due to the impact of a scale-up in large-scale preventive chemotherapy campaigns over the past decade. However, schistosomiasis transmission is not yet interrupted by the current control measure. Therefore, understanding clear limitations such as reinfection rates will highlight the need for an effective and sustainable schistosomiasis transmission control strategies.

- 1. Rationale

Despite the available information on the factors contributing to the rapid schistosomiasis reinfection rate, to our knowledge, there is no review which described the schistosomiasis reinfection rate based on the evidence gathered from different parts of the world. The available article reviewed data on *Schistosoma japonicum* reinfection rate only in China (11). Hence, this study was designed to conduct a systematic review, with meta-analysis, of studies that estimated human schistosomiasis reinfection rate globally. A clear understanding of the schistosomiasis reinfection rate is crucial for planning effective and sustainable strategies to control schistosomiasis transmission.

- 1. Justification

Currently, the main control strategy for schistosomiasis is through chemotherapy with Praziquantel. Hence it is mandatory to estimate the reinfection rate of the disease and the attributing factors towards elimination.

- 1. Specification

Population: human participants of any age, sex, race and from any geographical area. Intervention: before follow-up, the study participants were diagnosed with any of human *Schistosoma* species and treated till cured. Control: not applicable in this review. Outcome: our outcome of interest was human schistosomiasis reinfection rate.

1. **Methods**
   1. Search strategy

Electronic data bases that will be searched and key search terms

We will search the following electronic databases: PUBMED, HINARI and Google Scholar. References To obtain study articles containing information of our interest, the following key search terms: “Schistosomiasis”, “*Schistosoma mansoni*”, “*Schistosoma haematobium*”, “*Schistosoma japonicum*” and “reinfection” joined using “OR” and “AND” booleans will be used to create search query. We will perform manual screening of the listed references in each selected articles to search for any other relevant articles. We will manually search the bibliographies of the selected articles for the presence of other related references.

- 1. Selection criteria

We included all studies presenting original research work with the following characteristics. Population: articles must have presented original research work conducted on human participants of any age, sex, race and from any geographical area. Intervention: the articles must have indicated that before follow-up, the study participants were diagnosed with any of human *Schistosoma* species and treated till cured. However there was no limitation on the type of drug, number of doses used and time interval from treatment to cure. Outcome: our outcome of interest was human schistosomiasis reinfection rate. The included articles must have presented data on the reinfection rate with any *Schistosoma* species. According to our definition, reinfection rate means the proportion of egg-positive participants who get cured (egg-negative by microscopic techniques) and then become egg-positive after a certain specified period of time. We excluded articles on experimental and non human studies, review articles, letter to editors and articles which did not report on reinfection rate as per our definition.

After combining search results from the three databases, all duplicates will be removed. Reviewer AZ will screen all searched articles based on the titles and abstracts for their eligibility to be included in full text review. Articles eligible for full text review will be retrieved. All accessed full text articles will be reviewed by two independent reviewers (AZ and VM) for the eligibility to be included in data extraction. Matched articles selected by the two reviewers will be subjected to data extraction; whenever there will be mismatch the third reviewer (TM) will be involved.

- 1. Quality assessment

Criteria that will be used to assess methodological quality

The study quality for selected studies will be assessed based on 9 criteria as described in the Joanna Briggs Institute critical appraisal checklist for use in reviews of prevalence studies (12). The criteria are the appropriateness of sample frame to address the target population, appropriateness of sampling methods of study participants, sample size, description of study subjects and setting, data analysis coverage of the identified sample, validity of methods used in identification of disease condition, standard and reliable measurement of the condition to all participants, appropriateness of statistical analysis and adequateness of the response rate and way used to manage low response rate.

Performance of quality assessment

Each criterion will be given either of the two options of YES if a criterion will be met or NO if a criterion will not be met. A YES option will be graded as 1 and NO option as 0. The minimum score of 0 will be given if all criteria will not be met and maximum score of 9 will be given if all criteria will be met. Studies with overall grades ranging from 0 - 4 will be considered of low quality, 5 - 7 moderate quality and 8 – 9 high quality. Studies with moderate to high quality will be included in the review. Two reviewers (AZ and VM) independently will assess the quality of selected articles.

- 1. Data extraction

Key data to be extracted

The key data that will be extracted include schistosomiasis reinfection rate, follow up time, type of *Schistosoma* species, geographical area (Country), participants age, sample size and study settings. Other data include journal name, name of first author, year of study, year of study publication, study design, diagnostic test used and treatment drug.

Performance of data extraction and presentation

Data will be independently extracted by two reviewers (AZ and VM) using a designed data extraction form. Prepared data extraction form will be pre-tested by three reviewers before use. When there will be difference in data extracted by the two reviewers from similar article, the third reviewer (TM) will be invited to independently extract the data. Similar data extracted by either one of the first two reviewers and the third reviewer will be taken. When the third reviewer came with different data, consensus will be reached through discussion. Data will be presented in form of tables and forest plots.

- 1. Data synthesis

Data be combination

Statistical methods will be used to combine data. Statistical tests will be computed by using statistical R software.

Potential sources of effect heterogeneity and assessment

The potential sources of effect heterogeneity include sample size, participants age, geographical area, study settings, follow up time and *Schistosoma* species. These factors will be classified in several categories and assessed by using subgroup analysis.

1. **Process**
   1. Resources required and available to conduct the review

Relevant expertise: All authors had experience in teaching and research on medical parasitology and entomology. Authors had attended short courses on scientific writing and information searching.

Computing facilities: All authors had a laptop with internet access and ability to use computer software for data analysis

Research databases: All authors had access including account for PUBMED, HINDARI and/or Google Scholar

Bibliographic software: All authors had installed Mendeley on their laptops

Statistical software: All authors had installed statistical R and R studio software

- 1. Dissemination of review findings

Target audience: Scientific communities, neglected tropical diseases stake holders and the whole community

Publication type: Peer reviewed open access publication

Communication media: Poster and oral presentation in scientific conferences and workshops

1. **Timetable**

*Completion date Lead reviewer*

Draft protocol for internal review July 2019 Abdallah Zacharia

Protocol for external review July 2019 Abdallah Zacharia

Searching and study selection August – December 2019 Abdallah Zacharia

Data extraction January – April 2020 Vivian Mushi

Quality assessment May 2020 Vivian Mushi

Draft report for peer review June 2020 Twilumba Makene

Submit for publication July 2020 Abdallah Zacharia

Celebrate publication December 2020 All

**References**

1. Sturrock RF. The schistosomes and their intermediate hosts. Trop Med Sci Pract London Imp Coll Press 2001. 2001;7–83.

2. World Health Organization. Key facts [Internet]. Schistosomiasis. 2016 [cited 2020 May 5]. Available from: https://www.who.int/news-room/fact-sheets/detail/schistosomiasis

3. World Health Organization. Current estimated total number of individuals with morbidity and mortality due to Schistosomiasis haematobium and S. mansoni infection in Sub-Saharan Africa [Internet]. Schistosomiasis. 2017 [cited 2020 May 21]. Available from: https://www.who.int/schistosomiasis/epidemiology/table/en/

4. Bustinduy AL, Parraga IM, Thomas CL, Mungai PL, Mutuku F, Muchiri EM, et al. Impact of polyparasitic infections on anemia and undernutrition among Kenyan children living in a schistosoma haematobium-endemic area. Am J Trop Med Hyg. 2013 Mar;88(3):433–40.

5. Montresor A, Gabrielli AF, Chitsulo L, Ichimori K, Mariotti S, Engels D, et al. Preventive chemotherapy and the fight against neglected tropical diseases. Vol. 10, Expert Review of Anti-Infective Therapy. Europe PMC Funders; 2012. p. 237–42.

6. Campbell SJ, Savage GB, Gray DJ, Atkinson JAM, Soares Magalhães RJ, Nery S V., et al. Water, Sanitation, and Hygiene (WASH): A Critical Component for Sustainable Soil-Transmitted Helminth and Schistosomiasis Control. PLoS Negl Trop Dis. 2014;8(4).

7. Grimes JE, Croll D, Harrison WE, Utzinger J, Freeman MC, Templeton MR. The roles of water, sanitation and hygiene in reducing schistosomiasis:A review. Parasites and Vectors. 2015;8(1):1–16.

8. Doenhoff MJ, Cioli D, Utzinger J. Praziquantel: Mechanisms of action, resistance and new derivatives for schistosomiasis. Vol. 21, Current Opinion in Infectious Diseases. 2008. p. 659–67.

9. Wilkins HA. Reinfection after treatme t of schistosome infections. Parasitol today. 1989;5(3):83–8.

10. Mbanefo EC, Huy NT, Wadagni AA, Eneanya CI, Nwaorgu O, Hirayama K. Host determinants of reinfection with schistosomes in humans:A systematic review and meta-analysis. PLoS Negl Trop Dis. 2014 Sep;8(9):e3164.

11. Yang A, Nie Z, Chen F, Cai S, Liu Q, Guo Y. Meta-analysis of Schistosoma japonicum reinfection and its risk factors in Chinese population. Chinese J Epidemiol. 2015;36(2):181–5.

12. JBI. Critical appraisal checklist for prevalence studies. The Joanna Briggs Institute; 2017. 7 p.
